# Identification of a DNA Methylation-Driven Genes-Based Prognostic Model and Drug Targets in Breast Cancer: *In silico* Screening of Therapeutic Compounds and *in vitro* Characterization

**DOI:** 10.3389/fimmu.2021.761326

**Published:** 2021-10-20

**Authors:** Saisai Tian, Lu Fu, Jinbo Zhang, Jia Xu, Li Yuan, Jiangjiang Qin, Weidong Zhang

**Affiliations:** ^1^ School of Pharmacy, Second Military Medical University, Shanghai, China; ^2^ Department of Pharmacy, Tianjin Rehabilitation Center of Joint Logistics Support Force, Tianjin, China; ^3^ School of Pharmacy, Henan University, Kaifeng, China; ^4^ Cancer Hospital of the University of Chinese Academy of Sciences, Zhejiang Cancer Hospital, Hangzhou, China; ^5^ Institute of Basic Medicine and Cancer (IBMC), Chinese Academy of Sciences, Hangzhou, China; ^6^ Innovation Center of Chemical Biology, Institute of Interdisciplinary Integrative Medicine Research, Shanghai University of Traditional Chinese Medicine, Shanghai, China

**Keywords:** breast cancer, drug targets, DNA methylation, personalized prognostication, precision therapy

## Abstract

DNA methylation is a vital epigenetic change that regulates gene transcription and helps to keep the genome stable. The deregulation hallmark of human cancer is often defined by aberrant DNA methylation which is critical for tumor formation and controls the expression of several tumor-associated genes. In various cancers, methylation changes such as tumor suppressor gene hypermethylation and oncogene hypomethylation are critical in tumor occurrences, especially in breast cancer. Detecting DNA methylation-driven genes and understanding the molecular features of such genes could thus help to enhance our understanding of pathogenesis and molecular mechanisms of breast cancer, facilitating the development of precision medicine and drug discovery. In the present study, we retrospectively analyzed over one thousand breast cancer patients and established a robust prognostic signature based on DNA methylation-driven genes. Then, we calculated immune cells abundance in each patient and lower immune activity existed in high-risk patients. The expression of leukocyte antigen (HLA) family genes and immune checkpoints genes were consistent with the above results. In addition, more mutated genes were observed in the high-risk group. Furthermore, a *in silico* screening of druggable targets and compounds from CTRP and PRISM databases was performed, resulting in the identification of five target genes (HMMR, CCNB1, CDC25C, AURKA, and CENPE) and five agents (oligomycin A, panobinostat, (+)-JQ1, voxtalisib, and arcyriaflavin A), which might have therapeutic potential in treating high-risk breast cancer patients. Further *in vitro* evaluation confirmed that (+)-JQ1 had the best cancer cell selectivity and exerted its anti-breast cancer activity through CENPE. In conclusion, our study provided new insights into personalized prognostication and may inspire the integration of risk stratification and precision therapy.

## Introduction

Breast cancer (BRCA) is a female malignancy with the highest incidence worldwide, accounting for up to 11.7% of all cancer cases. (1). At the molecular level, breast cancer is a heterogeneous illness and could be categorized into four subtypes, namely, lumina A (cavity surface A), lumina B (cavity surface B), triple-negative & HER-2 positive breast cancer (2). In addition to systemic treatments including chemotherapy, endocrine therapy (hormone therapy), targeted therapy, and immunotherapy, surgical resection is the primary option for treating breast cancer. Early-staged, non-metastatic breast cancer is often curable. However, under currently available therapies, a full recovery of patients from advanced breast cancer with distant organ metastases is challenging (3). Thus, early detection, diagnosis, and effective therapies are necessary for improving the survival of breast cancer patients. Prognosis prediction of breast cancer has been exhaustively investigated over the past decade, and future therapy strategies will be more concerned with individualization and personalized medicine (4, 5).

Epigenetic changes are genetic changes that alter DNA methylation, gene expression, histone acetylation, and noncoding DNA (6). DNA methylation is a vital epigenetic change, and the site of DNA methylation is the addition of a methyl group to the 5’ site of a CpG (“cytosine preceding a guanosine”) (7). Methylation changes such as tumor suppressor gene hypermethylation and oncogene hypomethylation have critical occurrences in various cancers (8). In recent research, DNA methylation affects the prognosis of breast cancer dependent on molecular subtypes (9, 10), and is associated with the chemoresistance of breast cancer patients to standard therapy in clinical practice (11). Therefore, detecting DNA methylation-driven genes and examining their association with treatment outcomes could help predict the risk of breast cancer patients (12), finally leading to a more specialized clinical treatment. It was revealed that the identification of DNA methylation-driven genes is instrumental in understanding the process of cancer initiation (13), maintenance, and development (14). Recent studies have established a prognosis model on the basis of Bayesian network classification and applied it in classifying the test set into DNA methylation subgroups (15). More importantly, previous research have mostly concentrated on either gene or methylation expression data, without any integrated analyses. In addition, it is less often to tailor specialized management for high-risk patients with breast cancer. The development of treatment interventions is hampered by a lack of complete knowledge of the molecular as well as cellular processes that drive breast cancer.

To address the above challenges, for the first time, the present study identified DNA methylation-driven genes through integrating the transcriptomic as well as DNA methylation profiles of breast cancer. By using the random forest as a classifier, we further performed feature selection on DNA methylation-driven genes, designed a prediction model, and predicted potential therapeutic targets and agents for high-risk patients. We found that unique DNA methylation classifications may describe the heterogeneity of earlier breast cancer molecular subgroups and assist in the development of tailored remedies for new disease subtypes. Furthermore, five therapeutic target genes (HMMR, CCNB1, CDC25C, AURKA, and CENPE) and five drugs (oligomycin A, panobinostat, (+)-JQ1, voxtalisib, and arcyriaflavin A) were identified for those high-risk breast cancer patients, with the potential to improve current population-based therapeutic strategies in breast cancer management. Further *in vitro* experiment using high-risk prognostic sensitive drugs under different breast cancer cells showed that upregulated expression of the high-risk related gene CENPE was positively related with the sensitivity of breast cancer cells to (+)-JQ1. Our findings indicated that the methylation-driven gene-based model was reliable and can offer a novel approach for predicting and treating clinically high-risk patients and aid in improving BRCA molecular diagnostics and tailored treatments.

## Materials and Methods

### Patients and Samples

The mRNA raw count profiles of the TCGA-BRCA project and relevant clinical information were downloaded from the GDC data portal (https://portal.gdc.cancer.gov). A total of 1097 breast cancer patient samples with corresponding clinical information were available in TCGA. In addition, 888 DNA methylation profiles (Methylation450k, 790 tumor samples, and 98 non-tumor samples) of breast cancer patients were acquired from the UCSC Xena website (http://xena.ucsc.edu). Among 790 TCGA breast cancer DNA methylation samples, 785 samples included both RNA-sequencing data and paired DNA methylation data. In this paper, the gene methylation value was defined as the average DNA methylation value of all CpG sites in the promoters (transcription start sites (TSS) 1500 and TSS200) of a gene. An independent cohort (GSE86166 microarray dataset) with gene expression profiles of 366 breast cancer samples was downloaded from the Gene Expression Omnibus (GEO) database (https://www.ncbi.nlm.nih.gov/geo).

### Data Pre-Processing for DNA Methylation Profiles and Gene Expression Profiles

The methylation level of each probe was represented by the β value ranging from 0 (unmethylated) to 1 (fully-methylated). To simplify the DNA methylation data, probes with missing data in more than 50% of the samples were removed. The remaining probes with NAs were imputed by the K-nearest neighbor (KNN) imputation procedure. For raw count data from TCGA, the Ensembl IDs were transformed to gene symbols, and protein‐coding genes were selected. Next, we computed the transcripts per million (TPM) values, which showed a greater similarity to those generated from microarray analysis and higher comparability among samples. Then, TPM values were normalized using the log2(TPM+1) formula. For the GEO datasets, we normalized the expression datasets by Robust Multiarray Average with the R package “affy” (16). For multiple probes mapped to one gene, the mean value of expression was taken.

### Differentially Methylated Sites and Gene Analysis

In this study, the methylation analysis following chip analysis methylation pipeline (ChAMP) was performed with the required R package “IlluminaHumanMethylation450-kanno.ilmn12.hg19” as annotation (17, 18). Methylation β matrix was first filtered and imputed and then normalized through the embedded BMIQ method with the champ.filter function was applied to filter methylation probes with default parameters. The β value was normalized by the champ.norm function. Differentially methylated sites (DMSs) were detected by champ.DMP function. A probe was identified as a hypermethylated probe if the probe methylation level was greater than 0.3 in the tumor group but less than 0.2 in the normal group with an adjusted *P* value less than 0.05, and vice versa for hypomethylated probes. Furthermore, differentially methylated genes (DMGs) were determined according to probe location in corresponding genes. The average DNA methylation value for all CpG sites in the promoters (transcription start sites (TSS) 1500 and TSS200) of a gene was defined as the DNA methylation value for that gene.

### Comprehensive Analysis of Gene Expression and DNA Methylation

The MethylMix package in *R* was used to analyze integrated DNA methylation data of 785 breast cancer samples, 98 non-tumor samples, and paired gene expression data to identify DNA methylation events that significantly affect the expression of corresponding genes and to show that the gene was a DNA methylation-driven gene (19). For the MethylMix analysis, the correlation between the methylation data and paired gene expression data of DMGs in 785 breast cancer samples was determined to identify DNA methylation events that led to changes in gene expression, and only genes that met the correlation filter were recruited into further analysis. We also used a β mixture model to define a methylation state across a large number of patients, precluding the need for an arbitrary threshold. DNA methylation states between the 785 breast cancer samples and 98 corresponding non-tumor samples and was compared by conducting a Wilcoxon rank sum test. In this paper, significant q values of 0.05 calculated using P-value multiple testing correction with false discovery rate (FDR), and other parameters were set as default parameters.

### Generation and Validation of the Predictive Model

By using the random forest as a classifier, further feature selection was performed on DNA methylation-driven genes (20). Multivariate cox regression analyses were further conducted to evaluate relationships between the expression of the DNA methylation-driven genes and breast cancer prognosis and to detect the independent DNA methylation-driven genes significantly associated with the cancer prognosis. A risk score prediction model based on DNA methylation-driven genes was established through a linear combination of the expression levels of independent DNA methylation-driven genes using coefficients from multivariate Cox regression as weights. Breast cancer patients were stratified into low-risk and high-risk groups by the prediction model, with the optimal risk score as the cutoff point. Survival differences between high-risk patients and low-risk patients were evaluated by Kaplan-Meier survival plots and then compared by log-rank test. The GSE86166 dataset from the GEO database was used to validate the prognostic model. Whether the predictive power of the predictive model was independent of other clinical features of breast cancer patients was analyzed by univariate and multivariate Cox regression analyses.

### Analysis of Tumor Immune Signatures

In this paper, we calculated the tumor immune signatures from the following two aspects. Firstly, the levels of infiltrating immune and stromal cells were calculated by CIBERSORT (21), TIMER (22), MCP-counter (23), and quanTIseq (24) algorithms. Secondly, the expression of the leukocyte antigen (HLA) family genes and immune checkpoints genes also was calculated.

### Analysis of the Tumor Mutation Status

The information of somatic mutations in TCGA samples was downloaded from Genomic Data Commons Data Portal (https://portal.gdc.cancer.gov/). Concerning different mutation types, non-synonymous mutation variants and synonymous mutation variants are analyzed respectively in this paper. Differential mutated genes between the low- and high-risk groups were identified and the interaction effects of mutated genes also were analyzed by maftools package (25). In our analysis, only genes mutating more than 10 times in at least one group were considered.

### Cancer Cell Line Data

Expression profile data of human cancer cell lines were obtained from the Broad Institute Cancer Cell Line Encyclopedia (CCLE) project (https://portals.broadinstitute.org/ccle/) (26). The CERES scores of genome-scale CRISPR knockout screening of 18,333 genes in 739 cell lines were acquired from the dependency map (DepMap) portal (https://depmap.org/portal/) (27, 28). CERES score measures the dependency of the gene of interest in a specific cancer cell line, with a lower score indicating a higher possibility of the essentiality of the gene in cell growth and survival of the given cancer cell line. Drug sensitivity data of cancer cell lines were obtained from the Cancer Therapeutics Response Portal (https://portals.broadinstitute.org/ctrp) and PRISM Repurposing dataset (https://depmap.org/portal/prism/) (29, 30). The CTRP contains the sensitivity data of 481 compounds from over 835 cancer cell lines, and the PRISM contains the sensitivity data of 1497 compounds over from 480 cancer cell lines. Both two datasets provide area under the dose-response curve (AUC, area under the curve) values as a measurement of drug sensitivity, with a lower AUC indicating a higher sensitivity to treatment. In this study, compounds with more than 20% of missing data and cell lines derived from hematopoietic and lymphoid tissue were excluded. The KNN imputation was applied to impute the missing AUC values. Molecular data in CCLE were used for subsequent CTRP and PRISM analyses, as the cancer cell lines in both datasets were obtained from the CCLE project.

### Cell Lines and Drug Compounds

Human breast cancer cell line MDA-MB-231 and non-malignant breast epithelial cell line MCF10A were kind gifts from the Cancer Hospital of the University of Chinese Academy of Sciences (Zhejiang Cancer Hospital, Hangzhou China). The human breast cancer cell line MCF7 was purchased from American Type Culture Collection (ATCC). All the cell lines were maintained in RPIM 1640 medium (Gibco, CA, USA) supplemented with 10% fetal bovine serum (FBS, BI, Israel), 100 unit/mL penicillin, and 100 mg/mL streptomycin in a 5% CO_2_, 37°C incubator. Oligomycin A (Lot#: S147806), panobinostat (Lot#:120036), (+)-JQ1 (Lot#:S711013), voxtalisib (Lot#:120367), and arcyriaflavin A (Lot#:E0817) were commercially purchased from Selleck (USA).

### Cell Viability Assay

Cell counting kit-8 assay (CCK-8, Beyotime, China) was performed to detect the sensitivity of the breast cancer cell lines to the drugs (31). In short, when the cells were in good condition and growing exponentially, the culture medium was discarded and the cells were washed with PBS and incubated with trypsin for 3 minutes. The reaction was then terminated with the serum-containing culture medium to prepare cell suspension. Cells (1×10^4^ cells/well) were inoculated into 96-well plates, cultivated overnight to adhere to the wall. All five compounds were dissolved in DMSO at various concentrations and added to the 96-well plates for 24 h. CCK-8 assay was performed to determine cell viability according to the instructions and to assess the sensitivity of breast cells to the drugs. The IC_50_ values were calculated by GraphPad Prism 8.

### Quantitative Real-Time Polymerase Chain Reaction

The mRNA expression levels of methylated-gene CENPE in the MCF10A, MDA-MB-231, and MCF7 cell lines were examined. The primer sequence used for PCR is F: 5’-GATTCTGCCATACAAGGCTACAA -3’, R: 5’-TGCCCTGGGTATAACTCCCAA -3’. Briefly, the total RNA samples were obtained from all three cell lines using RNAiso Plus (lot# 9108, Takara, Japan) according to the manufacturer’s instruction. 5× Prime Script TM RT Master Mix (Perfect Real Time, lot# AJ21979A, Takara, Japan) was used to reverse-transcribe 20 uL RNA into the cDNA. The relative gene expression was quantified by the Universal SYBR Green Master (lot# 50837000, Roche, Switzerland). The Applied Biosystems (USA) was used for PCR amplification. The data were calculated with the 2^-△△^Ct.

### Flow Cytometry

When cells increased exponentially and had normal cell morphology, PBS was used washed and trypsin digestion. Next, cell suspensions were seed in 12-well plates. Cell state was observed and incubated with various concentrations of drug solution. Cultivated 24h, single-cell suspensions were prepared and incubated with APC Annexin V apoptosis Detection Kit with 7-AAD (lot#: 640930, BioLegnd, USA) for 15min, followed by flow cytometry analysis (32).

### Statistical Analysis

R software was used for statistical analysis of all the data. Survival analysis was performed using the R package survival, and Kaplan-Meier plots and log-rank tests were conducted to assess the difference in overall survival (OS) between the two groups (33). GSEA (gene set enrichment analysis) was performed using the package clusterProfiler in R (34). The Chi-square test was used to verify the association between categorical clinical information and defined groups. The Wilcoxon test (Mann–Whitney test) was used for continuous data. For all statistical analyses, a *P* value less than 0.05 was considered statistically significant.

## Results

### Identification of DNA Methylation-Driven Genes in Breast Cancer

**Figure 1:**
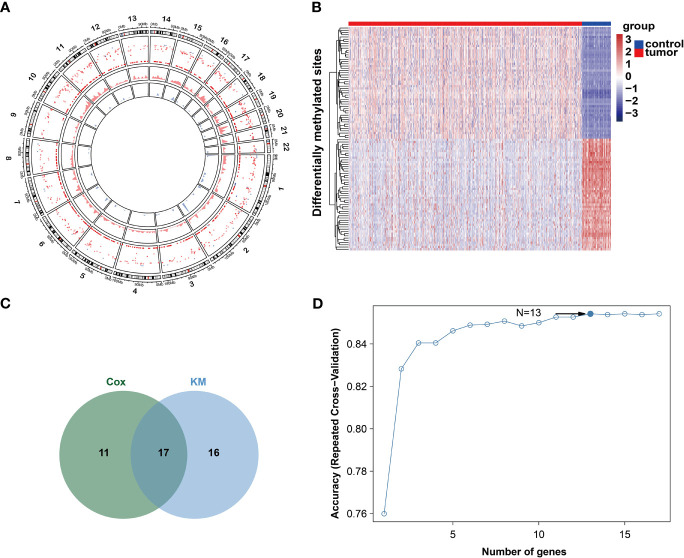
DNA methylation character in gene promoters. **(A)** Circos analysis of the hyper- and hypo DMSs between tumor and normal groups. The outer layer represents CpG sites heatmap. Red color represents CpG sites are hyper-methylated. Blue color represents CpG islands are hypo-methylated. The middle layer represents density plot of hyper-methylated CpG site. The inner layer represents density plot of hypo-methylated CpG site. **(B)** Heatmap of top50 hyper- and hypo DMSs. **(C)** Venn diagram of two methods. **(D)** Relation between classification accuracy and selected genes *via* recursive feature elimination algorithm.

The workflow of this study is shown in **Figure S1**. Under the previously mentioned thresholds, we detected a total of 2415 upregulated CpG sites and 134 downregulated sites. The detailed differentially methylated CpG sites are shown in **Supplementary Table S1**. The circle plot of differentially methylated sites is shown in **Figure 1A** and the top 100 sites heatmap is shown in **Figure 1B**. These differentially methylated sites were mapped onto corresponding genes. A gene was defined as differentially methylated if there is a differentially methylated CpG site on its promoter. After integrating the mRNA expression of genes, we finally obtained 1135 differentially methylated genes (DMGs, **Supplementary Table S2**).

As DNA methylation-driven genes play an important role in the initiation and progression of breast cancer, we further identified the DNA methylation-driven genes in this disease. Firstly, the DNA methylation and gene expression data of DMGs (DNA methylation data of 785 breast cancer samples and 98 non-tumor samples and the paired gene expression data of 785 breast cancer samples) were selected for further analysis. Then, using the MethylMix package, 194 DNA methylation-driven genes in total were screened (**Supplementary Table S3**). To examine the relationship between methylation-driven genes and the prognosis of breast cancer, the univariate cox proportional hazard regression analysis was performed and identified 28 statistically significant (*P* < 0.05) genes and the Kaplan-Meier survival analysis detected 33 statistically significant (*P* < 0.05) genes (**Supplementary Tables S4-5**). Finally, 17 intersect genes were obtained for further analysis (**Figure 1C**). The feature selection was conducted by recursive feature elimination (RFE) with a random forest as the classifier and a 10-fold cross-validation method in R package caret (35). We obtained an accuracy of 0.854 with 13 DNA methylation-driven genes (**Figure 1D**). These 13 DNA methylation-driven genes were AKR1E2, LIMD2, STAC2, SLC7A4, MAP4K1, PLAT, CXCL1, NACAD, RBP1, SFRP1, TOX, OSR1, and UBA7. The correlation analysis showed that the expression of these DNA methylation-driven genes had a significantly negative correlation with the level of DNA methylation (**Figures S2A–M**), showing a negative regulatory relationship between DNA methylation and gene expression.

### Generation and Validation of the Prognostic Model Based on DNA Methylation-Driven Genes

**Figure 2:**
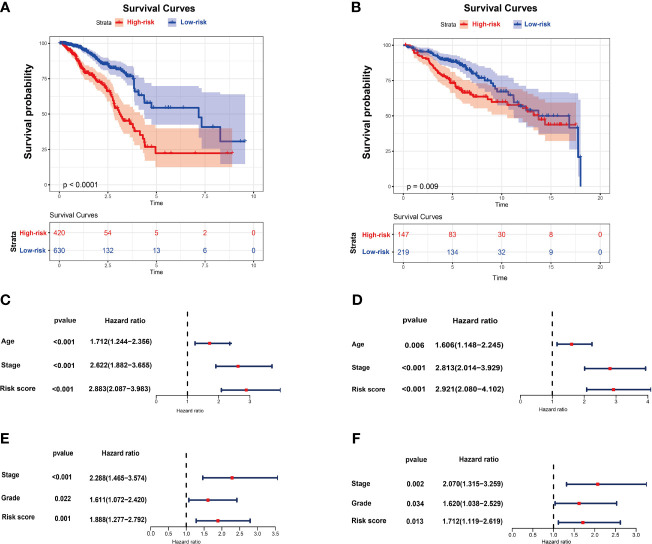
The risk score performance in the TCGA and GEO datasets. **(A)** The survival plot of OS in TCGA. **(B)** The survival plot of OS in GSE86166. **(C)** Univariate Cox regression analyses of the risk score and clinicopathological parameters in TCGA. **(D)** Multivariate Cox regression analyses of the risk score and clinicopathological parameters in TCGA. **(E)** Univariate Cox regression analyses of the risk score and clinicopathological parameters in GSE86166. **(F)** Multivariate Cox regression analyses of the risk score and clinicopathological parameters in GSE86166.

By using recursive feature elimination (RFE) with the random forest as a classifier and a 10-fold cross-validation method in R package caret, we obtained 13 DNA methylation-driven genes. A prognostic model was established with the regression coefficient from a multivariate Cox proportional hazard model. The risk score was calculated according to the formula: 0.1944 × AKR1E2 expression level + 0.0335 × SFRP1 expression - 0.0930 × LIMD2 expression - 0.0644 × STAC2 expression - 0.0965 × SLC7A4 expression level - 0.0042 × MAP4K1 expression level - 0.0932 × PLAT expression level - 0.1366 × CXCL1 expression - 0.0340 × NACAD expression - 0.0644 × RBP1 expression -0.1161 × TOX expression -0.0767 × OSR1 expression -0.1658 × UBA7 expression. The risk score was normalized to 0 to 1. The breast cancer patients were stratified into high-risk and low-risk groups according to the optimum cutoff point. The high-risk patients showed markedly worse OS than those with low risk (**Figure 2A**). The predictive performance of the prognostic model was further evaluated using 366 breast cancer samples with OS time and survival status in the validation dataset (GSE86166). The patients in the validation dataset were classified into low-risk and high-risk groups utilizing the earlier mentioned formula based on the optimal cutoff value. Consistent with the above findings, in the validation set patients in the high-risk group showed a markedly shorter median OS than those in the high-risk group (**Figure 2B**). To investigate whether the prognostic model was independent of the clinicopathological features, the univariate and multivariate Cox regression analyses were performed on the TCGA dataset. The results of univariate Cox regression demonstrated that the risk score was significantly associated with OS (high-risk of death group vs. low-risk of death group, HR =2.883, 95% CI 2.087-3.983, *P* < 0.01, **Figure 2C**). Additionally, in multivariable Cox regression, the risk score also showed a significant relationship with OS (high-risk of death group *v.s.* low-risk of death group, HR = 2.921, 95% CI 2.080-4.102, *P* < 0.01, **Figure 2D**). The same analysis was performed on the validation dataset with similar results (**Figures 2E-F**). These results indicated that the prognostic ability of the prognostic model was independent of other clinical features.

### Identification of Potential Drug Targets and Biological Processes for High Risk Score Breast Cancer

**Figure 3:**
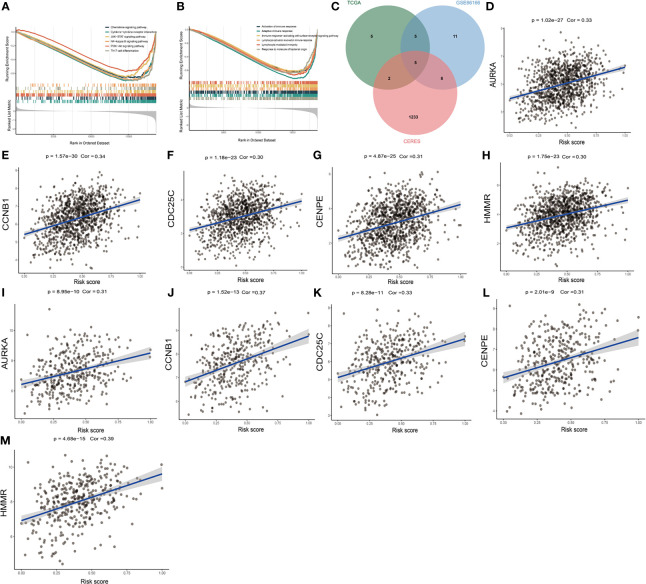
Identification of risk score-related biological processes and drug targets. **(A)** The enriched KEGG gene sets. **(B)** The enriched biological process gene sets. **(C)** Venn diagram of three datasets. **(D–H)** The relationship between gene expression and risk score of AURKA, CCNB1, CDC25C, CENPE, and HMMR genes in TCGA dataset. **(I–M)** The relationship between gene expression and risk score of AURKA, CCNB1, CDC25C, CENPE, and HMMR genes in GSE86166 dataset.

Transcriptome-based gene set enrichment analysis (GSEA) was conducted to determine the pathways and biological processes involved in breast cancer patients with different risks. We found that “Cytokine-cytokine receptor interaction”, “Chemokine signaling pathway”, “PI3K-Akt signaling pathway”, “JAK-STAT signaling pathway”, “Th17 cell differentiation”, and “NF-kappa B signaling pathway” were significantly enriched (*P* < 0.05) (**Figure 3A** and **Table 1**). In addition, the GO enrichment results showed that many biological processes associated with immunity were enriched (**Figure 3B** and **Table 2**), which was consistent with the KEGG results. These results indicated that the DNA methylation-driven genes-based risk score could influence these pathways and predict the survival of breast cancer patients.

**Table 1:** The enriched KEGG pathways associated with the signature.

ID	Description	Set size	Enrichment score	NES	pvalue
hsa04060	Cytokine-cytokine receptor interaction	293	-0.709	-1.842	0.000
hsa04062	Chemokine signaling pathway	190	-0.662	-1.702	0.000
hsa04151	PI3K-Akt signaling pathway	352	-0.572	-1.494	0.000
hsa04630	JAK-STAT signaling pathway	162	-0.654	-1.680	0.000
hsa04659	Th17 cell differentiation	105	-0.706	-1.796	0.000
hsa04064	NF-kappa B signaling pathway	102	-0.699	-1.776	0.000

**Table 2:** The enriched biological process associated with the signature.

ID	Description	Set size	Enrichment score	NES	pvalue
GO:0002250	Adaptive immune response	411	-0.722	-1.893	0.000
GO:0002449	Lymphocyte mediated immunity pathway	253	-0.672	-1.751	0.000
GO:0002237	Response to molecule of bacterial origin	339	-0.643	-1.686	0.000
GO:0002285	Lymphocyte activation involved in immune response	183	-0.649	-1.681	0.000
GO:0002253	Activation of immune response	441	-0.624	-1.638	0.000
GO:0002429	Immune response-activating cell surface receptor signaling pathway	357	-0.614	-1.611	0.000

Proteins presenting a high positive correlation with risk score might have potential therapeutic effects on high-risk patients. However, most of the human proteins lack obvious active sites to which small molecule compounds can bind or have been found in cells where are inaccessible for biological agents. With the purpose of identifying potentially druggable therapeutic targets for high-risk patients, we collected data of 2249 drug targets of 4484 compounds and conducted a two-step analysis for screening candidate targets (36) (**Supplementary Table S6**). Firstly, the correlation coefficient between the expression of druggable proteins and the risk score was calculated, and 17 protein targets were found to have a correlation coefficient higher than 0.30 (P < 0.05) in the TCGA dataset. Similarly, 29 protein targets were identified from GSE86166 and ten genes, including HMMR, CCNB1, CDK1, CCNA2, CDC25C, P4HA1, PAICS, AURKA, CENPE, and RAD51 were identified in both datasets mentioned above. Finally, the correlation analysis between the CERES score and the risk score based on breast cancer cell lines was conducted. Notably, CDK1, CCNA2, P4HA1, PAICS, and RAD51 showed a CERES score higher than zero in most breast cancer cell lines, indicating that they might not be essential to breast cancer. The remaining five genes HMMR, CCNB1, CDC25C, AURKA, and CENPE were considered to be potential therapeutic targets, and inhibiting the functions of these genes in high-risk patients could potentially achieve a favorable treatment efficacy (**Figure 3C**). The correlation figures of these five genes in TCGA and GSE86166 datasets are shown in **Figures 3D-M**.

### The Relationship Between Risk Score and BRCA Immune Signature

**Figure 4:**
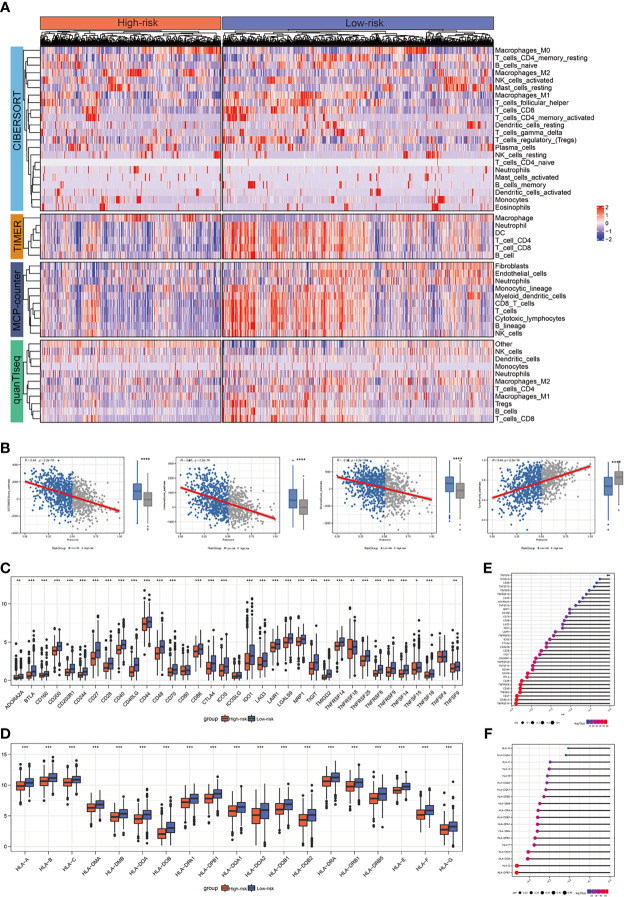
The correlation between risk score and expression of the immune checkpoints/HLA family genes. **(A)** Landscape of immune and stromal cell infiltrations in the low- and high-risk patients. **(B)** Association between estimate score, immune score, stromal score, tumor purity, and risk score and their distribution in the low- and high-risk groups. **(C)** Analyses for the expression of immune checkpoints genes in different groups. **(D)** Analyses for the expression of HLA family genes in different groups. **(E)** Correlation analysis for risk score and expression of immune checkpoints. **(F)** Correlation analysis for risk score and expression of HLA family genes. *P < 0.05; **P < 0.01; ***P < 0.001; ns, not significant.

In this research, using CIBERSORT, TIMER, MCP-counter, and quanTIseq algorithms, the abundance of infiltrating immune cells between two groups was calculated and the results demonstrated that most of the immune and stromal cells decreased in the high-risk group (**Figure 4A**). We also calculated the estimate score, immune score, stromal score and tumor purity by using estimate package for each sample. According to the result, we found that estimate scores, immune scores and stromal scores were significantly lower in high-risk patients, while tumor purity was higher (**Figure 4B**). We further investigated gene expression of the 35 immune checkpoints and 19 HLA family genes between the high-and low-risk patients. According to the Wilcoxon test, 32 immune checkpoints and all HLA family genes were significantly modulated in the high-risk group (**Figures 4C-D**). In addition, our analysis also showed that the risk score had a strong negative correlation with the expression of 18 immune checkpoints and 16 HLA genes, including TNFRSF14, CD40LG, CD27, TMIGD2, HLA-DPB1, and HLA-E (**Figures 4E-F**).

### Mutation Status in BRCA Patients in the High- and the Low-Risk Groups

**Figure 5:**
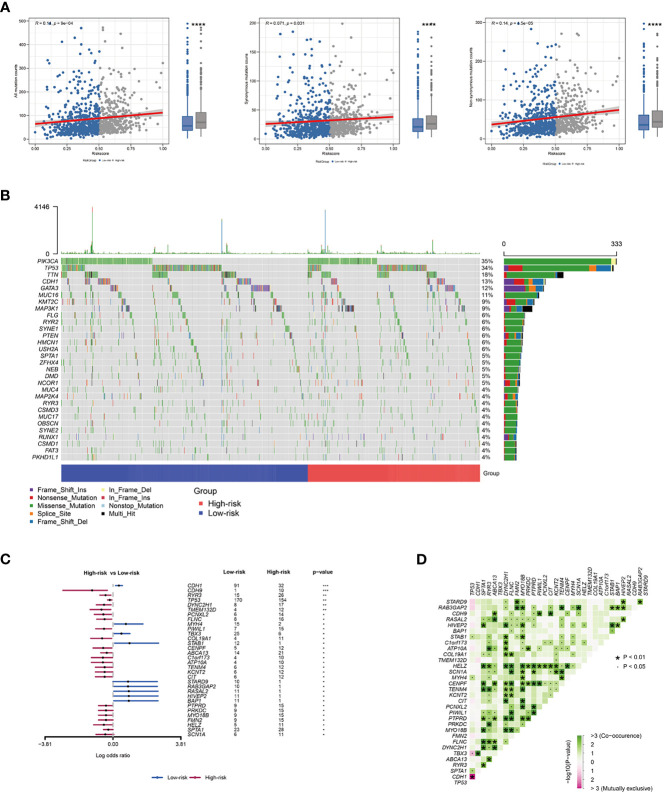
The relationship between risk score and BRCA immune signature. **(A)** Association between all mutation counts, synonymous mutation counts, non-synonymous mutation counts and risk score and their distribution in the low- and the high-risk groups. **(B)** Landscape of mutation status in all samples. **(C)** Forest plot of genes mutating differentially in patients of different groups. **(D)** Interaction effect of genes mutating differentially in different groups. *P < 0.05; **P < 0.01; ***P < 0.001.

In this paper, we downloaded somatic mutations from the TCGA database to investigate risk score-related mechanisms in BRCA. In the mutant frequency level, we found that more somatic mutations were observed in the high-risk group, including non-synonymous and synonymous mutations **(** **Figure 5A** **)**. The waterfall plots of mutation in all samples were shown in **Figure 5B**. Meanwhile, differential mutant genes also were calculated and 23 genes mutated more frequently in BRCA patients in the high-risk group, such as TP53, CDH9, RYR3, DYNC2H1, and TMEM132D (**Figure 5C**). However, only 9 genes mutated more frequently in BRCA patients in the low-risk group. Moreover, these differential mutant genes also show significant co-occurring or mutually exclusive mutation patterns (**Figure 5D**), which could shed lights on therapeutic strategies for breast cancer patients.

### Estimation of Drug Response in Clinical Samples

The CTRP and PRISM datasets, which contain the gene expression profiles and drug sensitivity profiles of hundreds of cancer cell lines can be utilized to construct the prediction model of drug response. We excluded compounds with NAs from more than 20% of the samples and cell lines derived from hematopoietic and lymphoid tissues. 680 cancer cell lines with 354 compounds in the CTRP dataset and 480 cancer cell lines with 1285 compounds in the PRISM dataset were recruited into the subsequent analysis. The pRRophetic package that had a built-in ridge regression model was used to predict the drug response of clinical samples based on their expression profiles, and an estimated AUC value of each compound in each clinical sample was obtained (36).

**Figure 6:**
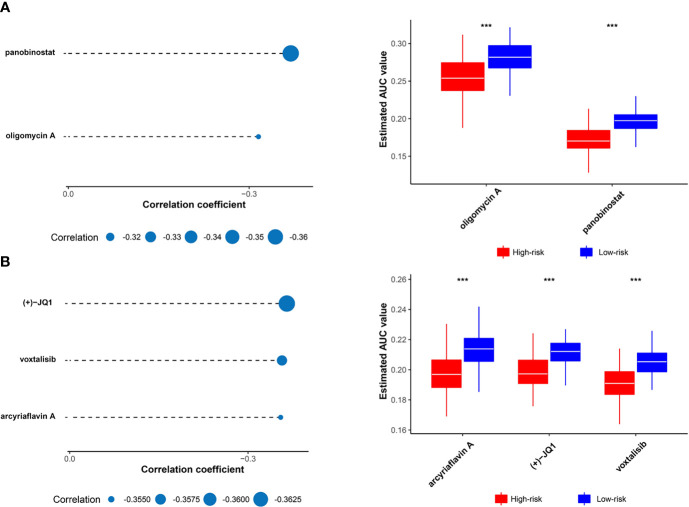
Identification of candidate drugs with higher drug sensitivity in high risk score patients. **(A)** The results of Spearman’s correlation analysis and differential drug response analysis of two CTRP-derived compounds. **(B)** The results of Spearman’s correlation analysis and differential drug response analysis of three PRISM-derived compounds. Note that lower values on the y-axis of box plots imply greater drug sensitivity. The statistical significance of pairwise comparisons is annotated with symbols in which *** represent *P* ≤ 0.001.

Two different approaches were adopted to identify the candidate with higher drug sensitivity in high-risk patients. The analyses were performed using CTRP- and PRISM-derived drug response data, respectively. First, differential drug response analyses between high-risk score (top decile) and low-risk score (bottom decile) groups were conducted to identify compounds with lower estimated AUC values in the high-risk score group (log2FC > 0.10). Next, Spearman correlation analysis between AUC value and the high-risk score was used to select compounds with a negative correlation coefficient (Spearman’s r < -0.30 for CTRP or -0.35 for PRISM). The analyses detected two CTRP-derived compounds (oligomycin A and panobinostat) and three PRISM-derived compounds ((+)-JQ1, voxtalisib, and arcyriaflavin A). All these compounds showed lower estimated AUC values in the high-risk group and a negative correlation with risk score (**Figures 6A, B**). Therefore, these compounds may have therapeutic effects on high-risk breast cancer patients.

### Upregulated Expression of CENPE in Breast Cancer Cells

**Figure 7:**
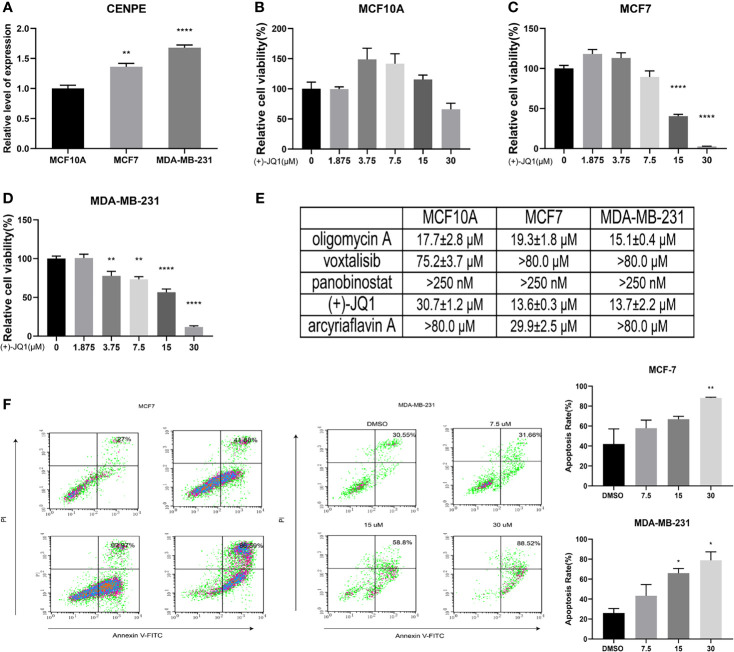
Biological verification of the association between methylation-driven genes and potential compounds. **(A)** The mRNA expression levels of CENPE in different cell lines. **(B)** The effects of (+)-JQ1 on the viability of MCF10A cells. **(C)** The effects of (+)-JQ1 on the viability of MCF7 cells. **(D)** The effects of (+)-JQ1 on the viability of MDA-MB-231 cells. **(E)** The IC_50_ values of selected compounds in different cell lines. **(F)** The effects of (+)-JQ1 on apoptosis in MCF7 and MDA-MB-231 cell lines. *P < 0.05; **P < 0.01; ****P < 0.0001.

We identified five potential target genes HMMR, CCNB1, CDC25C, AURKA, and CENPE for patients with high-risk scores. Among them, the CENPE gene encodes a centromere binding protein and mitotic kinesin, which has been demonstrated as a promising target for cancer drug development (37). We further examined the mRNA expression level of CENPE in breast epithelial cell line MCF10A and breast cancer cell lines MDA-MB-231 and MCF7. The results showed that CENPE was significantly elevated in breast cancer cell lines (**Figure 7A**).

### *In vitro* Anti-Breast Cancer Activity of Selected Drugs

To explore the sensitivity of breast cancer cells to the selected drugs, we examined the effects of all five compounds on the viability of MCF10A, MCF7, and MDA-MB-231 cell lines. The results showed that only (+)-JQ1 had selective cytotoxicity for breast cancer cells (**Figures 7B–E** and **Figure S3**). (+)-JQ1 showed comparable cytotoxicity in MCF7 and MDA-MB-231 cell lines, with IC_50_ values of 13.57 μM and 13.72 μM, respectively. However, MCF10A cells (IC_50_ = 30.74 μM) were much less sensitive to (+)-JQ1 than MCF7 and MDA-MB-231 cell lines. We further examined the effects of (+)-JQ1 on breast cancer cell apoptosis. The results showed that with the increased concentration of (+)-JQ1, the compound induced apoptosis in both breast cancer cell lines (**Figure 7F**). Taken together, our results indicated that (+)-JQ1 might exert its anti-breast cancer activity through CENPE.

## Discussion

As one of the most common malignant cancers, breast cancer imposes serious health problems on women all over the world (38, 39). Considerable efforts have been devoted to the improvement of breast cancer treatment, but its mortality rate remains high and patients often develop resistance to chemical therapy (40). Recent studies have shown that breast cancer is accompanied by genetic changes and epigenetic abnormalities, and DNA methylation is the most common epigenetic change (41, 42). Therefore, it is necessary to identify the role of DNA methylation-driven genes in the diagnosis of breast cancer for improving the prognosis and treatment of breast cancer.

RNAseq and microarray technologies have provided opportunities for the discovery of new genes involved in the epigenetic modulation of breast cancer (43). TCGA data have demonstrated the significant diversity of genetic alterations in human cancer (44), but not all abnormalities will exert a biological effect on breast cancer or stimulate its development. It is also necessary to distinguish between epigenetic changes promoting malignant phenotype and alterations of “passenger” genes without any biological effect (45). Therefore, a model-based instrument (MethylMix) has been used to identify DNA methylation-driven genes that affect their expression. In the present study, we used a ChAMP pipeline to detect DNA methylation-driven sites and identified DMGs, according to the probe location in corresponding genes. Subsequently, the MethylMix package in R was applied for analyzing integrated data to identify DNA methylation-driven genes. We obtained 13 DNA methylation-driven genes using recursive feature elimination with the random forest as a classifier and a 10-fold cross-validation method in R package caret. Then, multivariate Cox regression analysis was conducted to generate a prognostic model system based on these DNA methylation-driven genes. The model successfully stratified patients with breast cancer into high-risk and low-risk groups. Specifically, our model showed a high performance in predicting the survival of high-risk patients, who had significant differences in OS. We also verified the robustness of the model in the GEO dataset, which showed that the model had high performance. To assess the independence of the prognostic model in predicting OS, univariate and multivariate cox regression analyses were conducted, and the results indicated an independent correlation of the prognostic with the OS of breast cancer patients. Overall, these results suggested the prognostic significance of the DNA methylation-driven genes-based model in predicting the OS of breast cancer patients and the model was independent of other clinical features.

The GSEA method was performed on the gene set from GO term and KEGG pathway and the values demonstrated that the DNA methylation-driven genes-based risk score could influence immune-related pathways, including “Cytokine-cytokine receptor interaction”, “Chemokine signaling pathway”, “PI3K-Akt signaling pathway”, “JAK-STAT signaling pathway”, “Th17 cell differentiation”, and “NF-kappa B signaling pathway”. Using four immune infiltration algorithms and estimate algorithm, we calculated the abundance of infiltrating immune cells in each patient and we found that the high-risk group presented higher tumor purity, lower levels of immune and stromal cell infiltration, lower immunogenicity than patients in the low-risk group. These results suggested that high-risk tended to present an immune-suppressed status. Lower immune activities, such as lower immune cell infiltration and downregulation of HLA-I and HLA-II expressions existed in the high-risk group, which may be associated with the downregulation of checkpoints. Maftools analysis also showed higher somatic mutation status in the high-risk group. More evidence showed that tumor mutational burden (TMB) has been a biomarker of immunotherapy response and patients can benefit from immunotherapy if they have higher TMB (46, 47). In this research, we found that patients in the high-risk group had higher tumor mutational counts. However, as discussed above, patients in the low-risk group presented a higher immune activity, suggesting that high TMB did not necessarily predict high immunogenicity. Further analysis showed that the mutation status may be the major reason for the high TMB in the high-risk group. Strikingly, the higher frequency of co-mutations was observed in differential mutant genes, indicating that co-occurrence mutation may lead to an unknown change in different group patients.

Risk score as a biomarker is also used for precision oncology to guide the targeted treatment. Based on the druggable targets from 6125 compounds, five potential therapeutic targets (AURKA, CCNB1, CDC25C, HMMR, and CENPE) were identified, and five agents (oligomycin A, panobinostat, (+)-JQ1, voxtalisib, and arcyriaflavin A) were screened from CTRP- and PRISM-derived drug response data for high-risk breast cancer patients.

Aurora kinase A (AURKA) as a regulator of asymmetric satellite cell divisions, is a tumor suppressor with a high frequency of inactivating mutations in many cancers and the AURKA-CDC25C axis is a novel target for treating colorectal cancer (48). Advanced study found that methylation of AURKA by the histone methyltransferase multiple myeloma SET domain protein reduces p53 stability and regulates cell proliferation and apoptosis in multiple tumor cells (49). CCNB1 (also named CYCB1) is widely used as a marker for cell proliferation (50). It also promotes DNA repair when cells suffer from DNA damage (51). Besides, CCNB1 decreases proliferation and S-phase cell proportion and increases apoptosis, senescence, and G0/G1-phase cell proportion in cancer (50). In hepatocellular carcinoma, translation of CCNB1 could promote proliferation, metastasis, and sorafenib resistance, Conversely, methylated CCNB1 may help reduce cancer invasion (52). CDC25C is a key protein for G2/M transition and mitotic entry (53) and determines cell survival (54). Therefore, the degradation of the CDC25C protein delays cell progression (55). Although there are no studies on CDC25C methylation, previous study showed that CpG methylation of CDC25C upstream gene, disheveled binding antagonist of beta catenin 2 (DACT2), sensitized nasopharyngeal cancer cells to paclitaxel and 5-FU toxicity by suppressing β-catenin/CDC25C signaling, which indicates the methylation of CDC25C may obtain the same effect (56). HMMR gene locates human chromosome 5q33.2-qter and encodes a cell-surface receptor for hyaluronan (RHAMM) that mediates motility in many cell types (57). However, hyaluronan not only mediates motility receptor (HMMR) but also encodes evolutionarily conserved homeostasis, mitosis, and meiosis regulator (58, 59). For example, the mutation or loss of HMMR in animals induces neurodevelopmental defects (60). HMMR intersects the process of the cell cycle that stops cancer metastasis (59). Moreover, the expression of HMMR is associated with the progression of cancer, such as RHAMM, and has been suggested as a prognostic factor and a potential therapeutic target for pancreatic ductal adenocarcinoma (PDAC) and pancreatic neuroendocrine (PNET) (61). Methylation level of HMMR strongly related to Head and Neck Squamous Cell Carcinoma (62).

CENPE is a centromere binding protein and mitotic kinesin (63). In previous research, CENPE was inhibited by several compounds that have entered Phase I and Phase II clinical trials and raised the possibility of the range of kinesin-based drug targets (64). Recent study found that CENPE expression is associated with its DNA methylation status in esophageal adenocarcinoma (65). In this study, we examined the relationship between CENPE expression and the anti-breast cancer activity of the selected compounds. First, we observed that CENPE mRNA expression level is significantly higher in breast cancer cell lines than that in normal breast epithelial cells. We also found that one of the compounds (+)-JQ1 selectively inhibited breast cancer cell viability while it did not show significant cytotoxicity against normal breast epithelial cells, suggesting that the expression of CENPE may enhance the sensitivity of breast cancer cells to (+)-JQ1. We also observed that (+)-JQ1 induced apoptosis in both breast cancer cell lines in a concentration-dependent manner, which further confirmed the significant anti-breast cancer activity of the compound.

The *in vitro* experiments were performed for evaluating these high-risk prognostic sensitive drugs, and it was found that the upregulated expression of high-risk related gene CENPE was accompanied by an increased sensitivity of breast cancer cells to (+)-JQ1, indicating that the methylation-driven model was successfully established. The high-risk model was reliable and can provide a new perspective for the prediction and treatment of clinically high-risk patients with breast cancer. However, some limitations in the present analyses should be equally noted. Firstly, the number of cohorts with both RNA-seq and DNA methylation data available was limited. Secondly, the conclusion was only drawn from *in silico* analysis, while the well-designed prospective population-based studies should be conducted for further verification. Finally, the results of drug target prediction and therapeutic agent prediction failed to support each other. Moreover, future experimental and clinical validations are necessary to better promote the clinical application of our findings.

## Conclusions

In conclusion, this study developed a novel gene model consisting of thirteen DNA methylation-driven genes and validated that the model system was strongly predictive of breast cancer prognosis. Our findings supported that genes regulated by DNA methylation were likely related to the treatment outcomes of cancer. The risk score model showed an important clinical significance in both low- and high-risk patients. For patients with low-risk scores, clinicians can adopt a low-toxicity therapy strategy to avoid ineffective over-treatment, while these patients can experience a better quality of life with a satisfactory prognosis. For those with high-risk scores, our study provided potential therapeutically effective targets and agents. Overall, the findings of this study offer new insights into personalized management of breast cancer prognosis and contribute to integrating personalized prognosis prediction with precision therapy.

## Data Availability Statement

Publicly available datasets were analyzed in this study. This data can be found here: https://portal.gdc.cancer.gov, http://xena.ucsc.edu, https://www.ncbi.nlm.nih.gov/geo, https://portals.broadinstitute.org/ccle, https://portals.broadinstitute.org/ctrp, https://depmap.org/portal/prism/, and https://depmap.org/portal/.

## Author Contributions

WZ and JQ designed the study. ST and JZ collected and analyzed the data. LF, JX, and LY analyzed the data and did *in vitro* experiment. ST and LF wrote the manuscript. WZ and JQ revised the manuscript. All authors contributed to the article and approved the submitted version.

## Funding

The work was supported by the National Key Research and Development Program of China [No. 2019YFC1711000, No. 2017YFC1700200], Professor of Chang Jiang Scholars Program, National Natural Science Foundation of China [grant numbers 82004003, 81520108030, 81973706], Shanghai Engineering Research Center for the Preparation of Bioactive Natural Products [grant number 16DZ2280200], the Scientific Foundation of Shanghai China [grant numbers 13401900103, 13401900101], the Shanghai Sailing Program (No. 20YF1459000) and the project of National Multidisciplinary Innovation Team of Traditional of Chinese Medicine (ZYYCXTD-202004).

## Conflict of Interest

The authors declare that the research was conducted in the absence of any commercial or financial relationships that could be construed as a potential conflict of interest.

## Publisher’s Note

All claims expressed in this article are solely those of the authors and do not necessarily represent those of their affiliated organizations, or those of the publisher, the editors and the reviewers. Any product that may be evaluated in this article, or claim that may be made by its manufacturer, is not guaranteed or endorsed by the publisher.

## Supplementary Material

Supplementary Figure 1 Schematic diagram of the study design.

Supplementary Figure 2 Regression analysis between gene expression and DNA methylation of thirteen DNA methylation-driven genes in the TCGA dataset. **(A–M)** The relationship between gene expression and DNA methylation of AKR1E2, LIMD2, STAC2, SLC7A4, MAP4K1, PLAT, CXCL1, NACAD, RBP1, SFRP1, TOX, OSR1, and UBA7 genes.

Supplementary Figure 3 The effects of selected compounds on the viability of breast epithelial cells and breast cancer cells. **(A)** The effects of oligomycinA, voxtalisib, panobinostat, and arcyriaflarin-a on the viability of MCF10A cells. **(B)** The effects of oligomycin A, voxtalisib, panobinostat, and arcyriaflavin A on the viability of MCF7 cells. **(C)** The effects of oligomycin A, voxtalisib, panobinostat, and arcyriaflavin A on the viability of MDA-MB-231 cells.
