# Supplementary figures and images for: Identification of a DNA Methylation-Driven Genes-Based Prognostic Model and Drug Targets in Breast Cancer: In silico Screening of Therapeutic Compounds and in vitro Characterization
Source: Front Immunol. 2021 Oct 20;12:761326. doi: 10.3389/fimmu.2021.761326 (PMC8567755; doi:10.3389/fimmu.2021.761326)

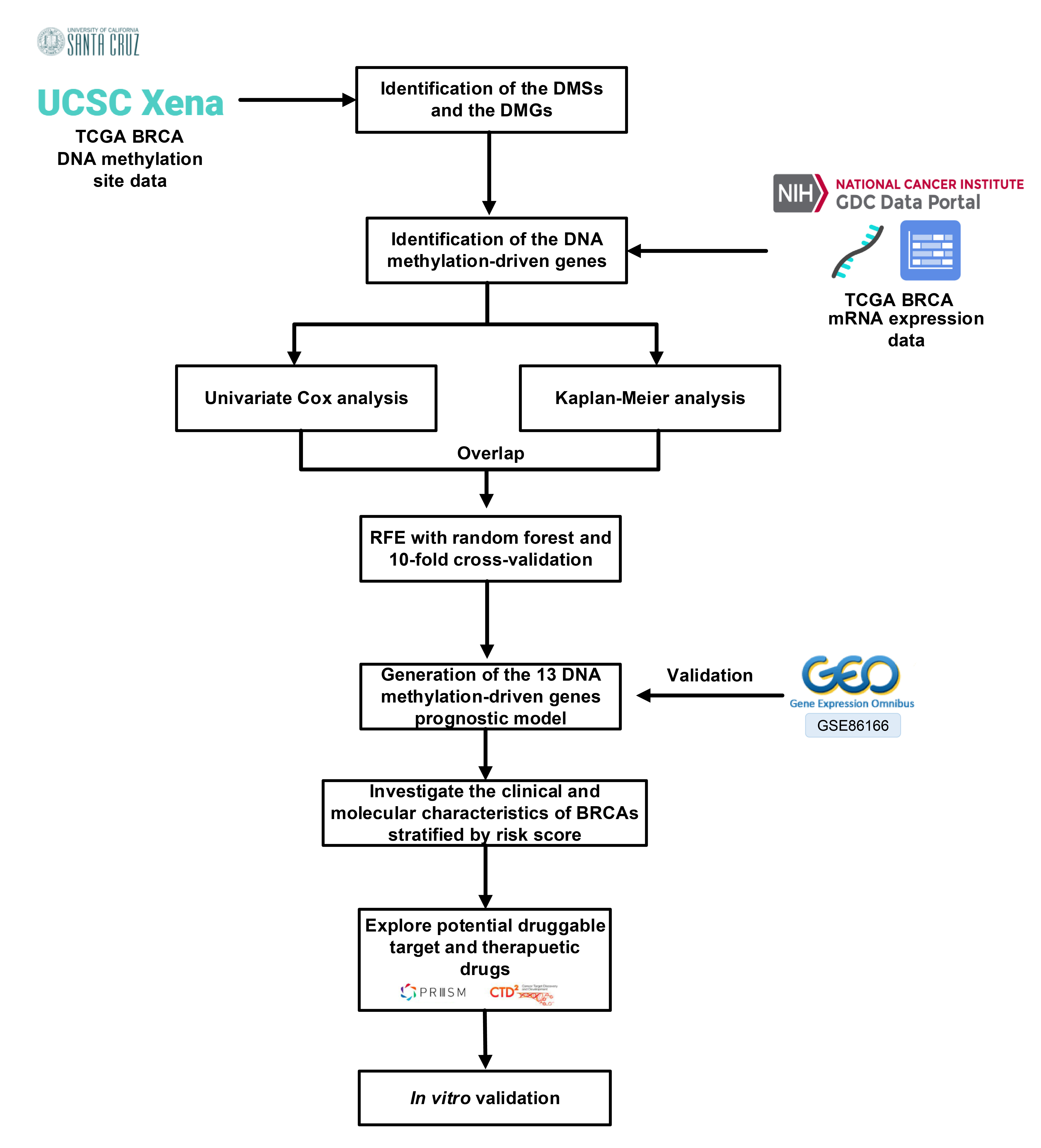

Supplement: Supplementary Figure 1 — Schematic diagram of the study design. [file Image_1.jpeg]

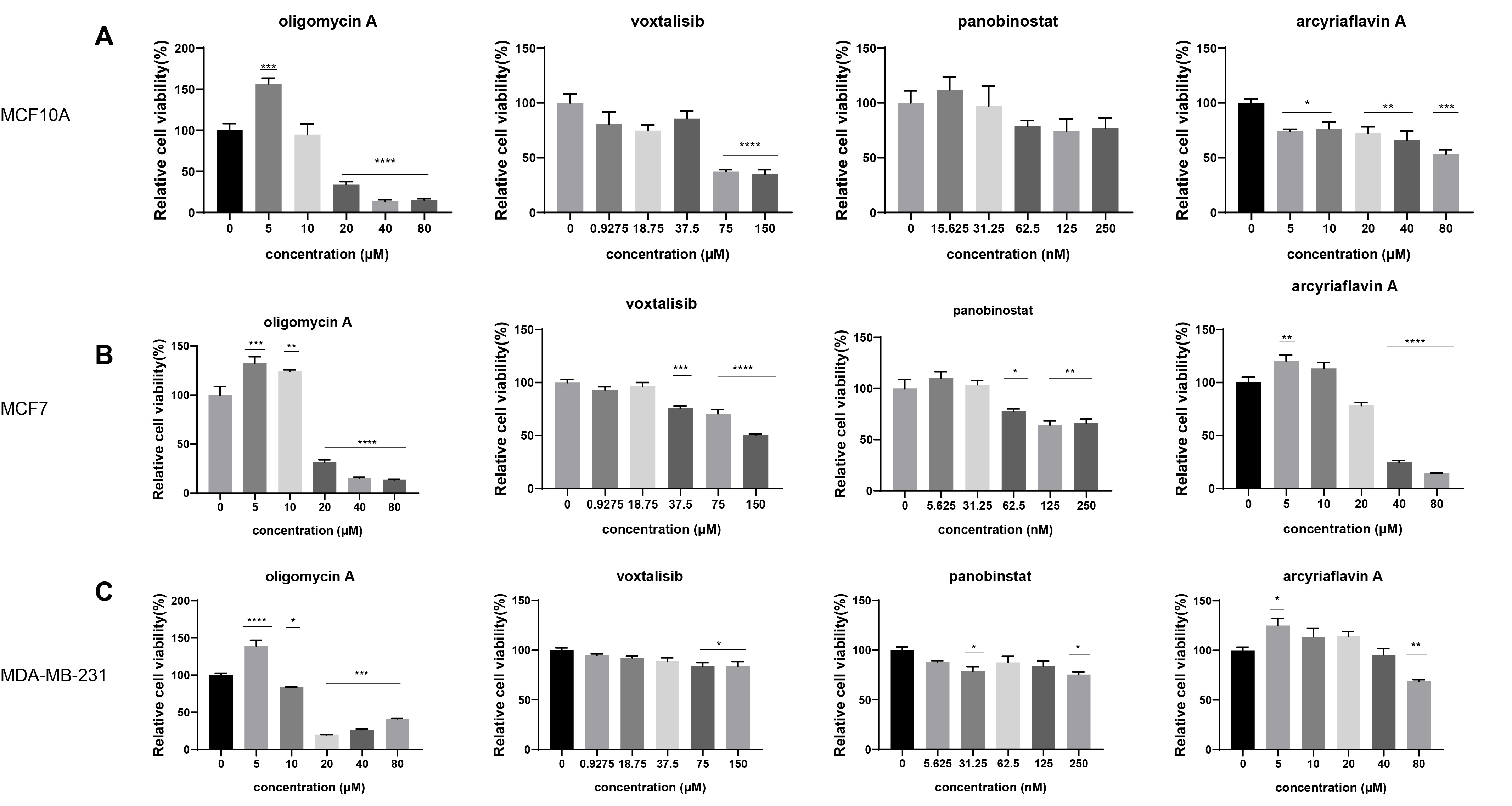

Supplement: Supplementary Figure 3 — The effects of selected compounds on the viability of breast epithelial cells and breast cancer cells. (A) The effects of oligomycinA, voxtalisib, panobinostat, and arcyriaflarin-a on the viability of MCF10A cells. (B) The effects of oligomycin A, voxtalisib, panobinostat, and arcyriaflavin A on the viability of MCF7 cells. (C) The effects of oligomycin A, voxtalisib, panobinostat, and arcyriaflavin A on the viability of MDA-MB-231 cells. [file Image_3.jpeg]
